# Supplementary material for: Classification of Clinical Outcomes in Hospitalized Asian Elephants Using Machine Learning and Survival Analysis: A Retrospective Study (2019–2024)
Source: Vet Sci. 2025 Oct 16;12(10):998. doi: 10.3390/vetsci12100998 (PMC12567809; doi:10.3390/vetsci12100998)
Supplement: Supplementary file 1 [file vetsci-12-00998-s001.zip › Supplementary figure captions.pdf]

Supplementary figure captions

Figure S1. Raw variable importance scores from the final weighted Random Forest model, showing all encoded predictor variables. Importance was derived from impurity-based measures across 500 trees. Categorical predictors (e.g., sex, disease group) were expanded into binary (dummy) variables during model training. LOS; Length of stay, EEHV; Elephant endotheliotropic herpesvirus.

Figure S2. One-vs-rest calibration plots for each class in the multiclass Random Forest classification model.

Figure S3. Confusion matrix of the final weighted multiclass Random Forest model, evaluated on the held-out test set (N = 94). True clinical outcomes (deceased, ongoing, recovered) are shown on the horizontal axis, and predicted classes on the vertical axis.

Figure S4. Calibration plot for the binary classifier (deceased vs. recovered). The dashed line indicates perfect calibration.

Figure S5. Multivariable cause-specific Cox regression results for hazard of death. Hazard ratios with 95% confidence intervals and *p*-values are shown. Female sex and integument disease group are reference categories. Model was stratified by cause-specific event, with recovery treated as a censoring event.

Figure S6. Multivariable cause-specific Cox regression results for hazard of recovery. Hazard ratios with 95% confidence intervals and *p*-values are shown. Female sex and integument disease group are reference categories. Model was stratified by cause-specific event, with death treated as a censoring event.

Figure S7. Cause-specific survival curves for death by disease system. Curves depict the cumulative probability of survival from death event over length of hospital stay by disease system. Shaded areas represent 95% confidence intervals.

Figure S8. Cause-specific survival curves for recovery by disease system. Curves show the cumulative probability of recovery across hospital days by disease system. Shaded areas represent 95% confidence intervals.
