# Supplementary material for: Classification of Clinical Outcomes in Hospitalized Asian Elephants Using Machine Learning and Survival Analysis: A Retrospective Study (2019–2024)
Source: Vet Sci. 2025 Oct 16;12(10):998. doi: 10.3390/vetsci12100998 (PMC12567809; doi:10.3390/vetsci12100998)
Supplement: Supplementary file 1 [file vetsci-12-00998-s001.zip › Table S1 Other breakdown.pdf]

Table S1. Full diagnostic breakdown of cases grouped under “Other” in the main classification model. This includes rare or poorly defined clinical conditions excluded from the top-level disease categories due to low frequency.

**Diagnosis (within “Other”)**	**N cases**	**% of “Other”**	**Outcome breakdown**
Tetanus	3	42.86%	Deceased: 2, Recovered: 1
Respiratory condition	2	28.57%	Recovered: 1, Ongoing 1
Adrenal insufficiency	2	28.57%	Deceased: 2
**Total “Other”**	7	100%	Deceased: 4, Ongoing: 1, Recovered: 2
