# Supplementary material for: Classification of Clinical Outcomes in Hospitalized Asian Elephants Using Machine Learning and Survival Analysis: A Retrospective Study (2019–2024)
Source: Vet Sci. 2025 Oct 16;12(10):998. doi: 10.3390/vetsci12100998 (PMC12567809; doi:10.3390/vetsci12100998)
Supplement: Supplementary file 1 [file vetsci-12-00998-s001.zip › Table S2 Schoenfeld residual tests.pdf]

Table S2. Schoenfeld residual tests for proportional hazards assumption in Cox regression.

Covariate	Chi-squared	df	P-value
Age	1.93	1	0.165
Sex	0.01	1	0.904
Disease group	27.32	10	0.002
GLOBAL	28.29	12	0.005
