# Supplementary material for: Classification of Clinical Outcomes in Hospitalized Asian Elephants Using Machine Learning and Survival Analysis: A Retrospective Study (2019–2024)
Source: Vet Sci. 2025 Oct 16;12(10):998. doi: 10.3390/vetsci12100998 (PMC12567809; doi:10.3390/vetsci12100998)
Supplement: Supplementary file 1 [file vetsci-12-00998-s001.zip › Table S3 Pre- and post-weighted model performance_edit.pdf]

Table S3. Random Forest (RF) model performance with 95% confidence interval (CI) comparison between pre- and post-weighted models.

Outcome	Pre-weighted RF			Post-weighted RF		
	Precision (95% CI)	Recall (95% CI)	F1 score (95% CI)	Precision (95% CI)	Recall (95% CI)	F1 score (95% CI)
**Deceased**	0.800 (0.25-1)	0.333 (0.08-0.64)	0.471 (0.15-0.74)	0.438 (0.21-0.71)	0.583 (0.29-0.88)	0.500 (0.24-0.71)
**Ongoing**	0.333 (0-1)	0.200 (0-0.68)	0.250 (0-0.62)	0.400 (0.10-0.71)	0.800 (0.333-1)	0.533 (0.17-0.80)
**Recovered**	0.862 (0.79-0.93)	0.962 (0.91-1)	0.909 (0.86-0.95)	0.928 (0.86-0.99)	0.821 (0.73-0.90)	0.871 (0.81-0.92)
**Overall**	Accuracy = 0.84<br>ROC AUC = 0.806 (0.78-0.93)			Accuracy = 0.79<br>ROC AUC = 0.827 (0.719-0.912)		

Metric	Pre-weighted RF	Post-weighted RF
Precision (macro)	0.67	0.59
Recall (macro)	0.50	0.74
F1 Score (macro)	0.54	0.64
