# Supplementary material for: Classification of Clinical Outcomes in Hospitalized Asian Elephants Using Machine Learning and Survival Analysis: A Retrospective Study (2019–2024)
Source: Vet Sci. 2025 Oct 16;12(10):998. doi: 10.3390/vetsci12100998 (PMC12567809; doi:10.3390/vetsci12100998)
Supplement: Supplementary file 1 [file vetsci-12-00998-s001.zip › Table S4 Binary model performance_edit.pdf]

Table S4. Binary (Deceased vs. Recovered) Random Forest (RF) classification performance with 95% confidence interval (CI) after class weighting.

Outcome	Precision (95% CI)	Recall (95% CI)	F1 Score (95% CI)
**Deceased**	0.333 (0.11-0.57)	0.600 (0.25-0.89)	0.429 (0.18-0.63)
**Recovered**	0.944 (0.89-0.99)	0.850 (0.77-0.92)	0.89 (0.83-0.94)
**Overall**	Accuracy = 0.74		
	ROC AUC = 0.869 (0.79-0.95)		

Metric	Weighted binary RF
Precision (macro)	0.33
Recall (macro)	0.60
F1 Score (macro)	0.43
