# Supplementary material for: Classification of Clinical Outcomes in Hospitalized Asian Elephants Using Machine Learning and Survival Analysis: A Retrospective Study (2019–2024)
Source: Vet Sci. 2025 Oct 16;12(10):998. doi: 10.3390/vetsci12100998 (PMC12567809; doi:10.3390/vetsci12100998)
Supplement: Supplementary file 1 [file vetsci-12-00998-s001.zip › Table S5 Day-0 model.pdf]

Table S5. Day-0 model (excluding length of stay as the variable) performance after class weighting with 95% confidence interval (CI). Precision and F1-score were not defined for Ongoing class due to zero predicted positive cases across bootstraps.

Outcome	Precision (95% CI)	Recall (95% CI)	F1 Score (95% CI)
**Deceased**	0.333 (0–0.803)	0.167 (0–0.417)	0.222 (0–0.462)
**Ongoing**	NA	0.00	NA
**Recovered**	0.831 (0.753–0.909)	0.949 (0.897–0.988)	0.886 (0.832–0.932)
**Overall**	Accuracy = 0.80		
	ROC AUC = 0.713 (0.582-0.829)		

Metric	Day-0 model
Precision (macro)	0.58
Recall (macro)	0.37
F1 Score (macro)	0.55
