# Supplementary figures and images for: Classification of Clinical Outcomes in Hospitalized Asian Elephants Using Machine Learning and Survival Analysis: A Retrospective Study (2019–2024)
Source: Vet Sci. 2025 Oct 16;12(10):998. doi: 10.3390/vetsci12100998 (PMC12567809; doi:10.3390/vetsci12100998)

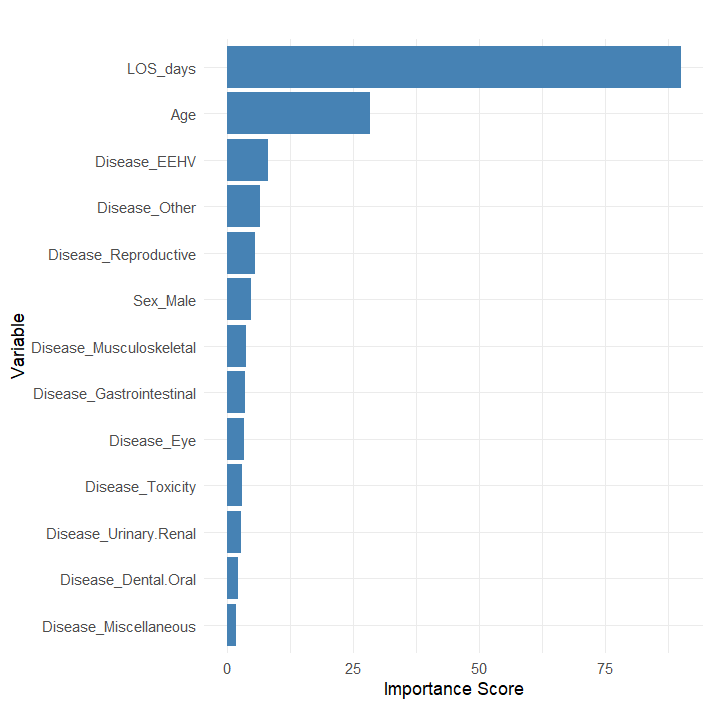

Supplement: Supplementary file 1 [file vetsci-12-00998-s001.zip › Figure S1.tiff]

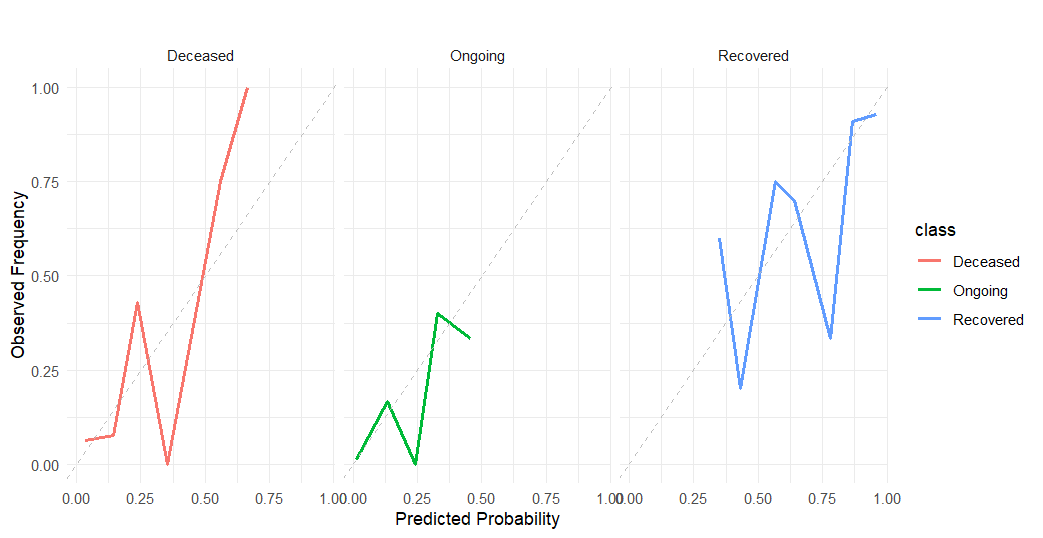

Supplement: Supplementary file 1 [file vetsci-12-00998-s001.zip › Figure S2.tiff]

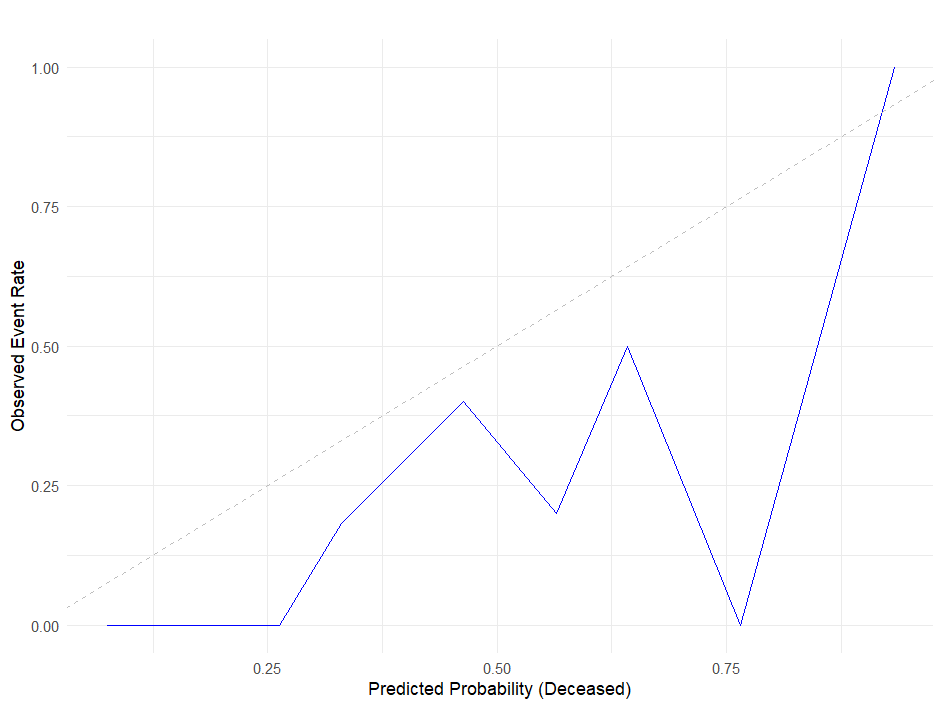

Supplement: Supplementary file 1 [file vetsci-12-00998-s001.zip › Figure S3.tiff]

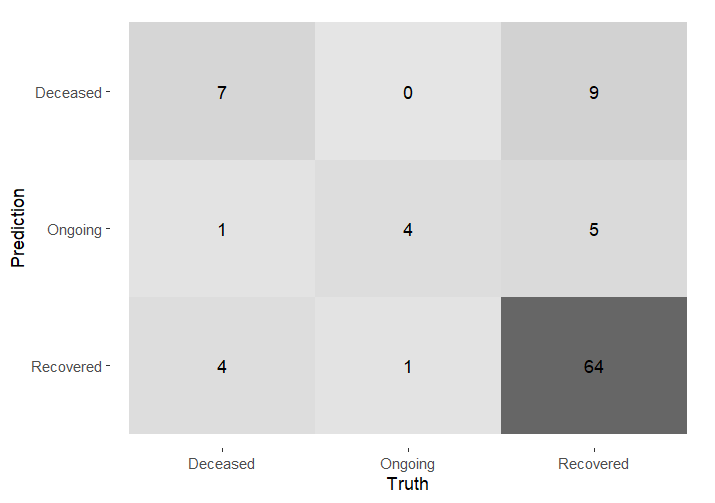

Supplement: Supplementary file 1 [file vetsci-12-00998-s001.zip › Figure S4.tiff]

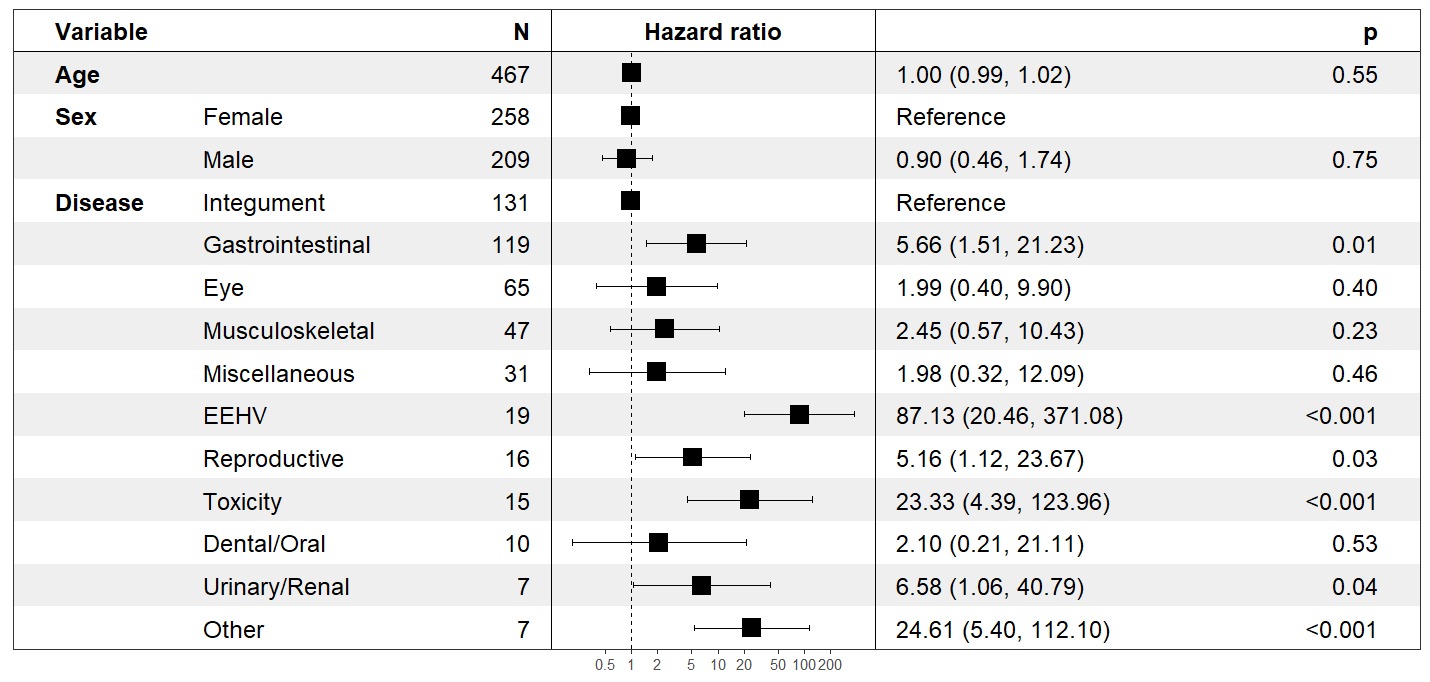

Supplement: Supplementary file 1 [file vetsci-12-00998-s001.zip › Figure S5.tiff]

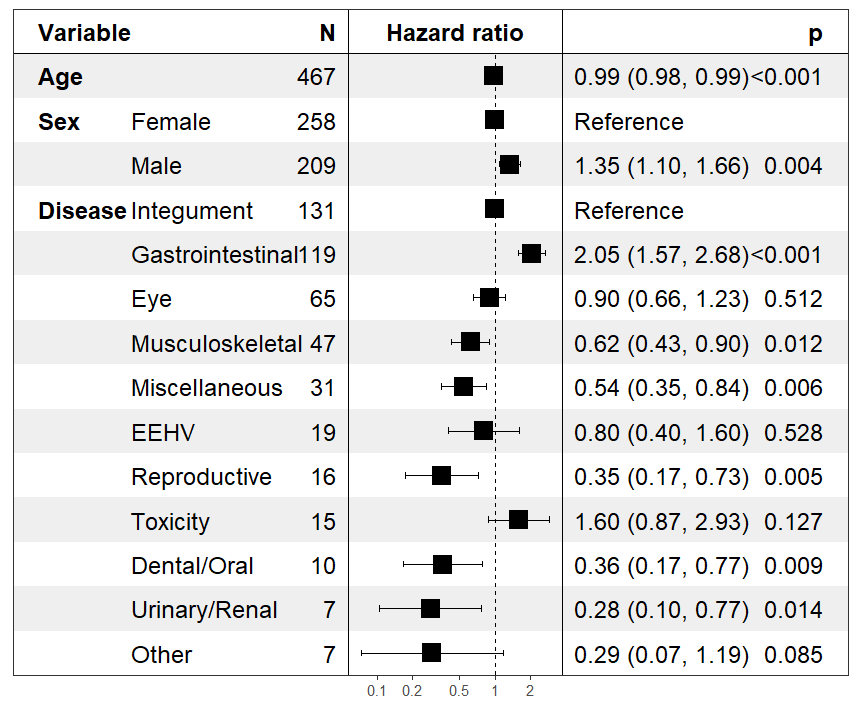

Supplement: Supplementary file 1 [file vetsci-12-00998-s001.zip › Figure S6.tiff]

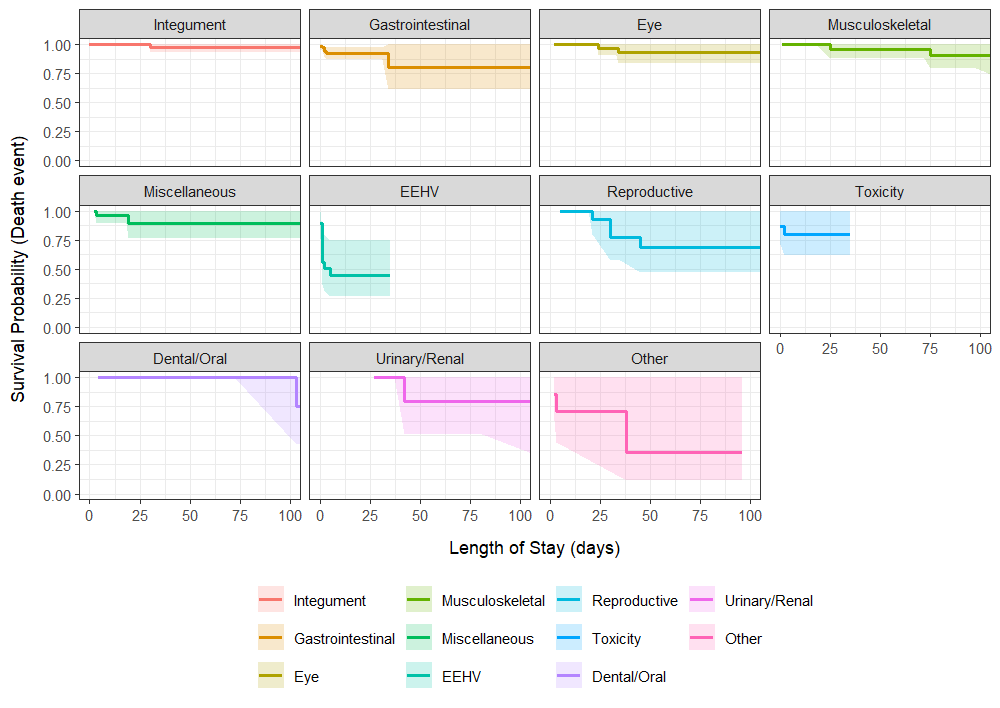

Supplement: Supplementary file 1 [file vetsci-12-00998-s001.zip › Figure S7.tiff]

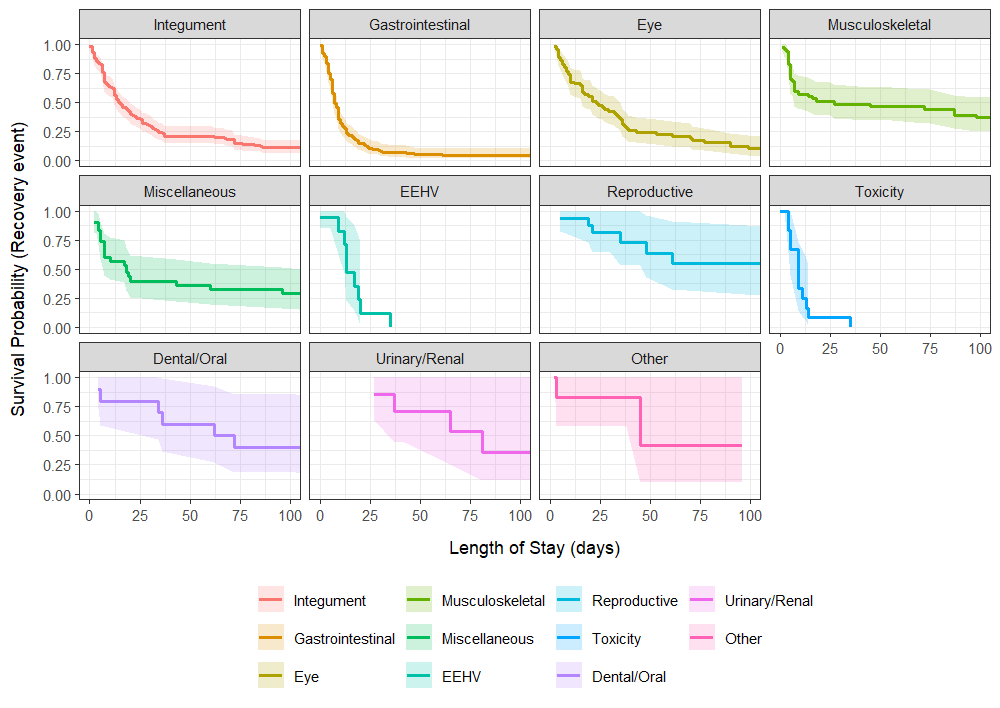

Supplement: Supplementary file 1 [file vetsci-12-00998-s001.zip › Figure S8.tiff]
