# Supplementary material for: Classification of Clinical Outcomes in Hospitalized Asian Elephants Using Machine Learning and Survival Analysis: A Retrospective Study (2019–2024)
Source: Vet Sci. 2025 Oct 16;12(10):998. doi: 10.3390/vetsci12100998 (PMC12567809; doi:10.3390/vetsci12100998)
Supplement: Supplementary file 1 [file vetsci-12-00998-s001.zip › Supplementary File 2 Elephant outcome classification tool.zip!/Elephant Prediction Model/User Instructions.pdf]

User Instructions for Elephant Prognostic Model (Excel + R Prediction Tool)

This tool allows elephant veterinarians to estimate treatment outcomes for hospitalized elephants based on four routine clinical variables. It provides predicted probabilities for each outcome category: **Deceased**, **Ongoing**, or **Recovered**, using a pre-trained machine learning model.

FILE in ZIP Package

File	Description
Elephant_Prognostic_Model_Prototype.xlsx	Excel file for entering input and viewing prediction results
Predict_Elephant_Outcome.R	R script to generate outcome predictions and write back to Excel
final_rf_weighted_model.rds	Pre-trained weighted Random Forest model
recipe_ml.rds	Preprocessing recipe used to transform inputs (dummy encoding, standardization, etc.)

Part 1: How to Fill in the Excel File

Open the file **Elephant_Prognostic_Model_Prototype.xlsx** and fill in the table as follows:

Column Name	Description
Elephant_ID	Unique ID (e.g., E001, CMU_2025_02)
Age	Age in years (numeric only)
Sex	Male or Female (case-sensitive)
Disease_Group	Must match one of the 12 categories listed below
LOS_Days	Number of days hospitalized (from admission to current date)

Important: Both Sex and Disease_Group must be typed exactly as shown below (case-sensitive, punctuation-sensitive).

Valid Disease_Group Categories:

Disease_Group	Descriptive
Integumentary	Skin wounds, abscesses, pressure sores, lacerations, or other skin-related problems.
Gastrointestinal	Colic, bloating, diarrhea, constipation, or digestive obstructions.
Eye	Cataracts, corneal ulcers, trauma, or infections affecting the eyes.
Musculoskeletal	Lameness, fractures, joint infections, or chronic orthopedic issues.
Miscellaneous	Non-specific signs (e.g., systemic weakness, vague inflammation) without clear diagnosis.
EEHV	Elephant Endotheliotropic Herpesvirus; hemorrhagic viral disease, mostly in calves.
Reproductive	Vaginal prolapse, dystocia, reproductive tract infections or tumors.
Toxicity	Ingestion of toxic plants, chemicals, or environmental toxins.
Dental/Oral	Broken tusks, gingivitis, tooth abscesses
Urinary/Renal	Cystitis, nephritis, kidney injury.
Other	Rare diseases (e.g., tetanus, respiratory failure, adrenal issues) grouped to avoid data sparsity.

Once the input is completed then click ‘save’ the Excel file.

Part 2: How to Run the Model

System Requirements:

- R (version $\geq$ 4.2.0)
- Required R packages (install only once):

```
install.packages(c("readxl", "openxlsx", "dplyr", "janitor", "forcats", "tidymodels"))
```

Step-by-Step Instructions:

1. Open **RStudio**.
2. Set your working directory to the folder containing the files.

Example R code:

```
setwd("C:/Your/Folder/Path")  # Replace with your actual path
```

3. Run the script:

```
source("Predict_Elephant_Outcome.R")
```

What the Script Will Do:

- Load and check the Excel input
- Process and transform data
- Predict outcome probabilities
- Results saved to: '**Elephant_Predictions_Output.xlsx**'
- Auto-populate results into 4 new columns:
  - Predicted_Deceased_Prob
  - Predicted_Ongoing_Prob
  - Predicted_Recovered_Prob
  - Predicted_Class (the most likely outcome)

Interpreting Results

After running the script, each row will have predicted probabilities (between 0 and 1) indicating the likelihood of each outcome:

Outcome	Meaning
Predicted_Deceased_Prob	Risk of death during hospitalization
Predicted_Ongoing_Prob	Chance of remaining under extended care
Predicted_Recovered_Prob	Chance of recovery and discharge
Predicted_Class	The outcome with the highest predicted probability

Example:

Elephant_ID	...	recovered	ongoing	deceased
E007	...	0.82	0.13	0.05

Interpretation: This elephant has a high chance of recovery (82%) and a low risk of death (5%).

Notes

- This model is trained from historical data at the National Elephant Institute Hospital and reflects those case patterns.
- It does not replace clinical judgment and should be used as a decision-support tool.
- The Ongoing category indicates that the elephant is still receiving treatment and may require extended hospitalization. It is not a final outcome.
- Input errors (e.g., misspelled fields) will cause the script to fail.

For technical issues, please contact:

Worapong Kosaruk

[worapong.kosa@cmu.ac.th, Faculty of Veterinary Medicine, Chiang Mai University]
